# Reply to Sumiyoshi, “Concerns about the interpretation of treatment effects”

**DOI:** 10.1128/aac.00723-24

**Published:** 2024-07-16

**Authors:** Xin Gu, Pingyu Zhou

**Affiliations:** 1 Institute of Sexually Transmitted Disease, Shanghai Skin Disease Hospital, School of Medicine, Tongji University, Shanghai, China; University of Fribourg, Fribourg, Switzerland

## REPLY

We thank Shougen Sumiyoshi (1) for commenting on our recent report (2) about the comparison on the effectiveness of ceftriaxone with aqueous crystalline penicillin G in treating ocular syphilis and for his suggestions that (i) our study should be based on a non-inferiority design, but the non-inferiority margin is not predefined and (ii) the standardized mean difference, rather than *P*-values, may be more appropriate to compare intergroup differences in a small sample size.

We agree that the results from studies with non-inferiority design and conclusions are more convincing. Because ocular syphilis is rare and difficult to diagnose, it is challenging for us to conduct prospective randomized, controlled clinical research with a large sample size. Acknowledging the limitations of retrospective studies is important in interpreting and applying our study results in both clinical and research contexts.

Following Shougen Sumiyoshi’s suggestion, we re-performed the statistical analysis of the raw data with the aid of a non-inferiority design and inference rule. To meet the statistical requirements of a non-inferiority design, we made the following definitions.

In the original article, we categorized the therapeutic effect into three levels (Effective, Improved, and Ineffective) to describe and show the respective therapeutic effects of the two groups. However, two categories of endpoints are more suitable for non-inferiority design. Therefore, we reclassified the endpoint into two groups (Effective and Ineffective). The effective group includes both the Effective and the Improved groups from the original classification.

The primary endpoint was the response rate after 3–6 months of treatment for ocular syphilis.

We did not define the non-inferiority margin before the study. Moreover, considering that this study was a retrospective, a sample size calculation was not performed. We referred to randomized, double-blind, non-inferiority trial of other antibiotic studies, where the non-inferiority margin ranged from 10% to 20% (3–5). Therefore, the non-inferiority margin was set at 15% in our study.

The primary efficacy endpoint was achieved by 28 (82.35%) of 34 patients in the ceftriaxone group and 110 (80.88%) of 136 patients in the penicillin group after propensity score matching (PSM), with a between-group difference of 1.47% (95% CI -12.94- 15.88%). Therefore we can also conclude that ceftriaxone efficacy is non-inferior to penicillin (Table 1）.

**TABLE 1:** Comparison of response rate in patients treated with ceftriaxone or penicillin after PSM

	Effective	Ineffective	Response rate (%)	Standard error (%)	95% CI (%)
Ceftriaxone	28	6	82.35	6.53	69.53–95.16
Penicillin	110	26	80.88	3.37	74.27–87.49
Difference			1.47	7.35	−12.94–15.88

In addition, Shougen Sumiyoshi suggested that the standardized mean difference (SMD), rather than the *P*-values, may be more appropriate to use when comparing intergroup differences in a small sample size study. SMD is commonly used statistic for assessing balance after PSM. However, in previous similar studies, the *P*-values have also been frequently used (6).

Overall, for a more comprehensive and accurate assessment of ceftriaxone’s effectiveness and safety, the next logical step involves conducting a multicenter, prospective, randomized, controlled trial.
